# Maternal Yes-Associated Protein Participates in Porcine Blastocyst Development via Modulation of Trophectoderm Epithelium Barrier Function

**DOI:** 10.3390/cells8121606

**Published:** 2019-12-11

**Authors:** Zubing Cao, Tengteng Xu, Xu Tong, Yiqing Wang, Dandan Zhang, Di Gao, Ling Zhang, Wei Ning, Xin Qi, Yangyang Ma, Tong Yu, Jason G. Knott, Yunhai Zhang

**Affiliations:** 1 Anhui Province Key Laboratory of Local Livestock and Poultry, Genetical Resource Conservation and Breeding, College of Animal Science and Technology, Anhui Agricultural University, Hefei 230036, China; zubingcao@ahau.edu.cn (Z.C.); xutengteng@ahau.edu.cn (T.X.); Tongxu@ahau.edu.cn (X.T.); wangyiqing@ahau.edu.cn (Y.W.); dandan@ahau.edu.cn (D.Z.); DiGao@ahau.edu.cn (D.G.); LingZhang@ahau.edu.cn (L.Z.); 18856899296@163.com (W.N.); 18720163@ahau.edu.cn (X.Q.); mayang890301@163.com (Y.M.); yt8504@ahau.edu.cn (T.Y.); 2 Developmental Epigenetics Laboratory, Department of Animal Science, Michigan State University, East Lansing, MI 48824, USA; knottj@msu.edu

**Keywords:** YAP, pig, blastocyst development, trophectoderm, tight junction

## Abstract

The establishment of a functional trophectoderm (TE) epithelium is an essential prerequisite for blastocyst formation and placentation. Transcription coactivator yes-associated protein (YAP), a downstream effector of the hippo signaling pathway, is required for specification of both the TE and epiblast lineages in mice. However, the biological role of YAP in porcine blastocyst development is not known. Here, we report that maternally derived YAP protein is localized to both the cytoplasm and nuclei prior to the morula stage and is then predominantly localized to the TE nuclei in blastocysts. Functionally, maternal *YAP* knockdown severely impeded blastocyst formation and perturbed the allocation of the first two lineages. The treatment of embryos with verteporfin, a pharmacological inhibitor of YAP, faithfully recapitulated the phenotype observed in *YAP* deleted embryos. Mechanistically, we found that maternal YAP regulates multiple genes which are important for lineage commitment, tight junction assembly, and fluid accumulation. Consistent with the effects on tight junction gene expression, a permeability assay revealed that paracellular sealing was defective in the trophectoderm epithelium. Lastly, *YAP* knockdown in a single blastomere at the 2-cell stage revealed that the cellular progeny of the YAP^+^ blastomere were sufficient to sustain blastocyst formation via direct complementation of the defective trophectoderm epithelium. In summary, these findings demonstrate that maternal YAP facilitates porcine blastocyst development through transcriptional regulation of key genes that are essential for lineage commitment, tight junction assembly, and fluid accumulation.

## 1. Introduction

Pigs are increasingly used as a dual-purpose model in agriculture and biomedical research [1,2,3]. The production of in vitro produced (IVP) embryos is an essential step involved in the generation of pigs for research. However, the developmental potential of pig IVP embryos is significantly lower compared to in vivo embryos and IVP embryos from other species [4,5,6,7,8,9]. The establishment of a functional trophectoderm (TE) epithelium is an essential prerequisite for blastocyst formation. Blastocyst formation is tightly regulated by TE-mediated exchange and accumulation of small molecules and water [10]. This characteristic is mainly mediated by the action of tight junction (TJ) complexes, ion gradient pumps, H_2_O channels, and cell polarity proteins that assemble on the TE apical and basolateral membranes [10,11]. Notably, functional studies revealed that the correct expression and localization of these proteins is required for blastocyst development [11,12]. However, the upstream signaling pathways that are responsible for regulating TE-specific features that are essential for blastocyst development in pigs remain largely unknown.

One such pathway that may have a role in the establishment of TE-specific features is the hippo signaling pathway. The hippo signaling pathway and its effector protein, yes-associated protein (YAP), play a key role in cell proliferation, survival, and differentiation in different cellular contexts [13]. YAP interacts with TEAD family proteins in the nucleus to promote transcriptional activation of target genes [14,15]. During mouse preimplantation embryo development, the hippo signaling pathway acts in a position dependent manner to govern the first cell fate decision (i.e., formation of TE and ICM) [16,17]. On the outside of the embryo, the hippo signaling pathway is inactive and YAP enters the nucleus to activate TE specific lineage genes (e.g., CDX2) and repress ICM specific genes (e.g., SOX2). In contrast, on the inside of the embryo hippo signaling is active and Lats Kinase phosphorylates YAP to prevent it from entering the nucleus and associating with TEAD4. Consequently, SOX2 expression is restricted to the inside cells, allowing formation of the pluripotent ICM [18]. Interestingly, a recent functional study that examined the roles of maternal and zygotic YAP in mouse preimplantation embryo development demonstrated that YAP is necessary for proper epithelization of the TE during blastocyst formation [18]. This observation led us to speculate that YAP might play a much broader role in blastocyst formation, beyond its established role in regulating lineage commitment.

In the present study, we evaluated the expression and function of YAP during porcine preimplantation development. We found that YAP is maternally expressed (i.e., oocyte derived) in pigs and its transcript is utilized during preimplantation embryo development. Using a combination of RNA interference (RNAi) and pharmacological approaches, we demonstrate that maternal YAP regulates the expression of key genes that are essential for tight junction (TJ) assembly, fluid accumulation, and lineage commitment. Disruption of these genes leads to defects in lineage allocation and TE paracellular sealing. Our findings provide new insights into the molecular mechanisms that regulate blastocyst formation in pigs.

## 2. Materials and Methods

### 2.1. Ethics Statement

Animal experiments were executed according to the Institutional Animal Care and Use Committee (IACUC) guidelines under current approved protocols at Anhui Agricultural University.

### 2.2. Preparation of Verteporfin and α-Amanitin

Verteporfin (MedChemExpress, HY-B0146) and α-amanitin (MedChemExpress, HY-19160) were separately dissolved in DMSO (Sigma, D2650) and stored at −20 °C. Embryo culture medium was used to dilute the two stock solutions to obtain the desired working solution. The same volume of DMSO was added into the medium as a control when the two chemicals were used.

### 2.3. Oocyte In Vitro Maturation

Ovaries were collected from a local slaughterhouse and transported to the laboratory at 28–35 °C in physiological saline solution. Follicular fluid was aspirated from medium-sized follicles at 3–6 mm in diameter. Cumulus-oocyte complexes (COCs) were selected under a stereomicroscope. Subsequently, COCs were cultured in one well of four-well plates containing 400 μL in vitro maturation medium (TCM-199 supplemented with 5% FBS, 10% porcine follicular fluid, 10 IU/mL eCG, 5 IU/mL hCG, 100 ng/mL l-cysteine, 10 ng/mL EGF, 0.23 ng/mL melatonin, 2.03 × 10^−5^ ng/mL LIF, 2 × 10^−5^ ng/mL IGF, 1.4 × 10^−5^ ng/mL FGF2, 100 U/mL penicillin, and 100 mg/mL streptomycin) for 42 h at 38.5 °C, 5% CO_2_, and 95% air with saturated humidity.

### 2.4. Parthenogenetic Activation (PA)

MII (metaphase II) oocytes were rinsed with activation medium (0.3 M mannitol supplemented with 0.1 mM CaCl_2_, 0.1 mM MgCl_2_, and 0.01% polyvinyl alcohol) three times and stimulated with single direct current (DC) pulse of 1.56 kV/cm for 80 μs using a cell fusion instrument (CF-150B, BLS, Hungary). Activated oocytes were then washed with porcine zygote medium 3 (PZM-3) and were incubated in chemically assisted activation medium (PZM-3 plus 10 μg/mL Cycloheximide and 10 μg/mL Cytochalasin B) for 4 h. Then, a group of 15 oocytes were cultured in 50 μL PZM-3 droplets at 38.5 °C, 5% CO_2_, and 95% air with saturated humidity.

### 2.5. In Vitro Fertilization (IVF)

Fresh mixed semen from two boars was washed three times with DPBS supplemented with 0.1% BSA, 75 μg/mL penicillin G, and 50 μg/mL streptomycin and spun at 1200 rpm, 17 °C for 3 min. Supernatant was removed after each centrifuge; spermatozoa pellets were resuspended with fertilization medium (mTBM supplemented with 2 mg/mL BSA and 2 mM caffeine) and were allowed to swim up for 1 h in a CO_2_ incubator. A group of 15 oocytes were incubated in 50 μL fertilization droplet at 38.5 °C and 5% CO_2_ saturated humidity. Sperm density was calculated and adjusted to a proper concentration. Sperm was then added to fertilization droplet containing oocytes and was co-incubated with oocytes at 38.5 °C and 5% CO_2_ saturated humidity for 5 h. Presumptive zygotes were washed with PZM-3 to remove excess sperm and were cultured for 7 days in PZM-3 medium at 38.5 °C, 5% CO_2_, and 95% air with saturated humidity. PZM-3 medium was replaced every 48 h during culture.

### 2.6. Microinjection

Three siRNA species were designed to target different sites of the porcine *YAP* coding region (GenePharma, Shanghai, China). Three siRNA species were dissolved and mixed together. siRNA was microinjected into the cytoplasm of MII oocytes, zygotes, and single blastomere of 2-cell embryos. For MII oocytes and zygotes, microinjection was performed in T2 medium (TCM199 plus 2% FBS) containing 7.5 μg/mL Cytochalasin B on a heating stage of an inverted microscope (Olympus, Japan). Approximately 10 pL siRNA solution (50 μM) was microinjected into cytoplasm of MII oocytes and zygotes. For single blastomere of 2-cell embryos, microinjection was only executed in T2 medium. 10 pL mixture of both YAP siRNA (100 μM) and mCherry mRNA (1408 ng/μL) was injected into cytoplasm of single blastomere of 2-cell embryos. Embryos were cultured in PZM-3 medium for 7 days. Information on sequences of the three YAP siRNA species used is listed in Supplementary Table S1.

### 2.7. In Vitro Transcription

mCherry mRNA that was used for microinjection was synthesized in vitro. pIVT-mCherry plasmids containing T7 promoter were linearized in preparation for in vitro transcription by digestion with BspQI. Linearized DNA templates were purified using a DNA clean & concentrator Kit (ZYMO RESEARCH, D4003, Tustin, CA, USA). In vitro transcription of mCherry mRNA was performed using the mMESSAGE mMACHINE T7 Kit (Ambion, AM1344, Shanghai, China) and the Poly (A) tailing Kit (Ambion, AM1350, Shanghai, China) according to the manufacturer’s manual. After in vitro transcription, mRNA was treated with TURBO Dnase to remove the DNA templates and was further purified using MEGAclear Kit (Ambion, AM1908, Shanghai, China). Purified mRNA was dissolved in RNase-free water. mRNA concentration was determined by a Nanodrop instrument (Thermo Scientific, Shanghai, China) and was aliquoted and stored at −80 °C.

### 2.8. Trophectoderm Permeability by the FITC-Dextran Exclusion Test

To investigate the effect of *YAP* knockdown on trophectoderm permeability, embryos from control and *YAP* knockdown group were cultured for 7 days. Blastocysts were then incubated in modified PZM-3 medium containing 1 mg/mL 40 kDa FITC-dextran (Sigma, FD40, St. Louis, MO, USA) for 40 min. Following the incubation, blastocysts were immediately washed and visualized under an inverted fluorescence microscope. Blastocysts that fluoresced green were classified as having impaired permeability.

### 2.9. Real-Time Quantitative Polymerase Chain Reaction (qPCR)

Total RNA was extracted from 10 oocytes or embryos using the RNeasy Mini Kit (Qiagen, 74104, Hilden, Germany) and was quantified by a Nanodrop instrument. RNA was then reversed into cDNA using a QuantiTect Reverse Transcription Kit (Qiagen, 205311, Hilden, Germany). cDNA was aliquoted and was stored at −80 °C until it was ready for use. The assembly of PCR was prepared in FastStart SYBR Green Master (Roche, 04673514001) and was run on StepOne Plus (Applied Biosystems). Three biological replicates were conducted for each gene. The primers that were used in this study are listed in Supplementary Table S2.

### 2.10. Immunofluorescence Staining

Oocytes or embryos were fixed in 4% paraformaldehyde solution for 15 min, permeabilized with 1% Triton X-100 in DPBS for 30 min at room temperature (RT), and were then blocked in DPBS containing 2% BSA at RT for 1 h. Samples were incubated in the blocking solution containing primary antibodies overnight at 4 °C. Following washing four times, the samples were incubated in the blocking solution containing secondary antibodies in the dark at RT for 1 h. After washing three times, the samples were counterstained for 10 min in 4,6-diamidino-2-phenylindole dihydrochloride (DAPI) or propidium iodide (PI) solution and were then loaded on glass slides followed by being covered with a glass coverslip. Finally, the samples were imaged using a laser scanning confocal microscope (Olympus, Japan). The specificity of commercially available primary antibodies that were used in this study was validated prior to usage (Supplementary Figure S1). The information regarding the primary and secondary antibodies that were used in this study is listed in Supplementary Table S3.

### 2.11. Western Blot

A total of 50 porcine embryos were collected in 10 μL lysis buffer (RIPA buffer supplemented with a cocktail of protease inhibitors) and were stored at −80 °C. Sample was then mixed with protein sample buffer (Beyotime, China) and heated at 95 °C for 5 min. Proteins were separated by SDS-PAGE with electrophoresis systems (Tanon, China) at 100 V for 120–150 min. The proteins were transferred to polyvinylidene fluoride (PVDF) membranes with electrophoretic transfer apparatus (Tanon, China) at 65 V for 120 min. Thereafter, membranes were blocked in blocking buffer (Beyotime, China) for 2 h and were then incubated with primary antibodies at 4 °C overnight. After washing three times, membranes were incubated with secondary antibodies for 1.5–2 h. Signals were detected with Lumi-Light Western Blotting Substrate (Roche) and images were acquired using the VersaDoc Imaging System (Bio-Rad). The signal intensity for bands was measured as the integrated intensity with Image J and was normalized to the background intensity. The detailed information on primary and secondary antibodies used in this study is listed in Supplementary Table S3.

### 2.12. Statistical Analysis

Data were analyzed using one-way ANOVA or Student’s *t* test (SPSS 17.0) and were presented as mean ± standard error of mean (mean ± S.E.M.). *p* < 0.05 was considered to be statistically significant.

## 3. Results

### 3.1. Developmental Expression of YAP mRNA and Protein in Porcine Early Embryos

**Figure 1:**
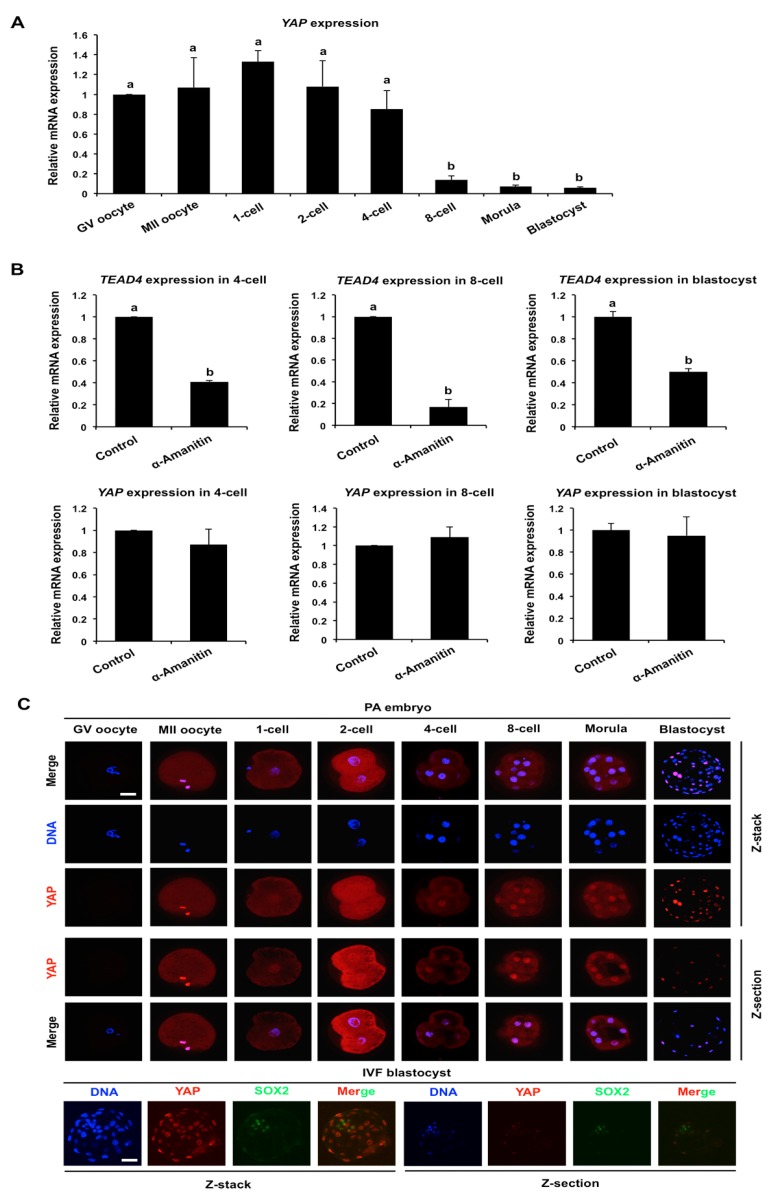
Expression of maternal YAP mRNA and protein in porcine early embryos. (**A**) Expression of *YAP* mRNA in oocytes and early embryos. Relative abundance of *YAP* mRNA was determined by qPCR. Data were normalized against endogenous reference gene *EF1α1* and the data from each stage were relative to GV oocyte. Data are shown as mean ± S.E.M and different letters on the bars indicate significant differences (*p* < 0.05). (**B**) Expression of *TEAD4* and *YAP* mRNA in embryos treated with or without α-amanitin. Relative abundance of *TEAD4* and *YAP* mRNA in 4-cell, 8-cell, and blastocysts was determined by qPCR. Data were normalized against endogenous reference gene *EF1α1* and the data from each stage were relative to the control group. Data are shown as mean ± S.E.M and different letters on the bars indicate significant differences (*p* < 0.05). (**C**) Expression and localization of YAP in oocytes and early embryos derived from Parthenogenetic Activation (PA) or In Vitro Fertilization (IVF). Oocytes and PA embryos at the indicated stages were stained for YAP (red) and DNA (blue). IVF blastocysts were double stained for YAP (red) and SOX2 (green). Representative Z-stack and Z-section images obtained by confocal microscopy are shown. The experiment was independently repeated three times with at least 20 oocytes or embryos per stage. Scale bar: 50 µm.

*YAP* mRNA expression and protein localization was previously reported in mouse oocytes and embryos [16,19], however, its expression and localization in porcine oocytes and early embryos has not been assessed. Real-time qPCR analysis was carried out to determine the expression pattern of *YAP* mRNA in porcine oocytes and early embryos. The results revealed that *YAP* mRNA was highly expressed in GV (geminal vesicle) oocytes and the levels persist up to the 4-cell stage. From the 8-cell stage onward, *YAP* mRNA levels gradually decreased and reached a minimum at the blastocyst stage (Figure 1A). To test whether *YAP* mRNA is strictly inherited from the oocyte, pools of embryos at the 2-cell, 4-cell, and morula stages were cultured for 24 h in the presence or absence of 25 μg/mL α-amanitin, a RNA polymerase II inhibitor. The next day control and treated embryos were collected at the 4-cell, 8-cell, and blastocyst stages and were subjected to qPCR to evaluate *YAP* mRNA expression. In parallel, the expression of *TEAD4* mRNA, a verified zygotic gene, was selected as a positive control for α-amanitin treatment (data not shown). As expected, the expression levels of *TEAD4* mRNA were significantly reduced in the α-amanitin treated embryos compared to those in the control group (*p* < 0.05). However, the expression levels of *YAP* mRNA did not change between the control and treatment groups (Figure 1B), indicating that *YAP* is a maternally derived transcript in early porcine embryos.

Next, the expression and localization of YAP protein was evaluated in porcine oocytes and embryos using immunofluorescence confocal microscopy. Oocyte and embryo images are represented as Z-stacks and Z-sections. We found that YAP protein was expressed in GV oocytes, MII oocytes, and at all stages of preimplantation development (Figure 1C). Interestingly, YAP protein was localized to both the cytoplasm and nucleus up until the morula stage (Figure 1C). During the morula to blastocyst transition, YAP protein became predominantly localized to the nuclei of TE cells, whereas SOX2 protein was localized to the nuclei of inner cell mass cells (Figure 1C). Collectively, these results demonstrate that *YAP* mRNA is maternally derived in porcine preimplantation embryos and its protein becomes enriched in the TE nuclei.

### 3.2. RNAi-Mediated Efficient Knockdown of Maternal YAP mRNA and Protein in Porcine Early Embryos

**Figure 2:**
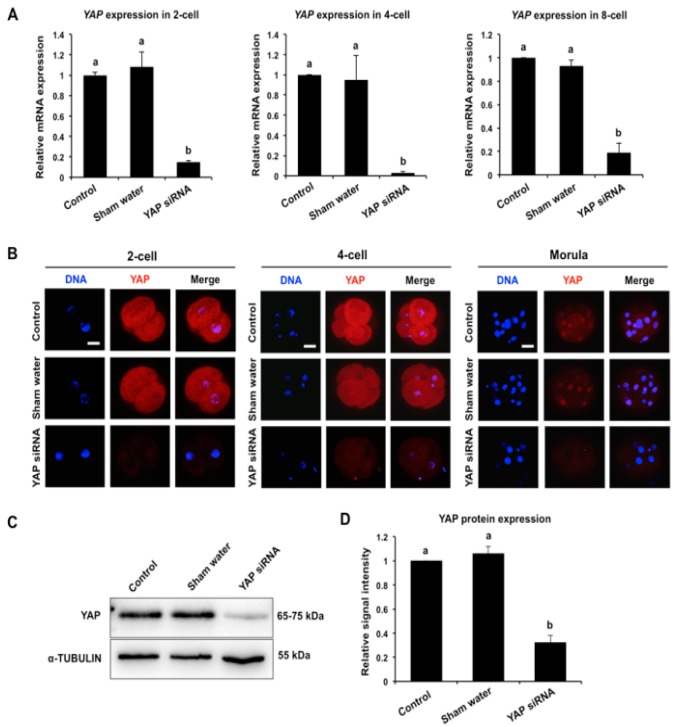
Validation of *YAP* knockdown efficiency in porcine embryos. (**A**) Expression levels of *YAP* mRNA in embryos. The relative abundance of *YAP* mRNA in 2-cell, 4-cell, and 8-cell embryos from control, sham water injection, and siRNA injection was determined by qPCR. Data were normalized against endogenous reference gene *EF1α1* and the data from each stage were relative to the control group. Data are shown as mean ± S.E.M and different letters on the bars indicate significant differences (*p* < 0.05). (**B**) Expression and localization of YAP protein in embryos. Two-cell, 4-cell, and morula stage embryos from each group were stained to indicate YAP (red) and DNA (blue). Representative images obtained by confocal microscopy are shown. The experiment was independently repeated three times with at least 24 embryos per stage. Scale bar: 50 µm. (**C**) Western blot analysis of YAP protein expression. Four-cell embryos from each group were used for western blot analysis and α-TUBULIN was used as a loading control. A representative image is shown. (**D**) Quantitative analysis of YAP protein expression using western blot. Data are expressed as mean ± S.E.M from three independent experiments and different letters on the bars indicate significant differences (*p* < 0.05).

To uncover the function of maternal YAP during porcine early embryo development, we utilized an RNAi approach to deplete *YAP* mRNA and protein. For this experiment, MII oocytes were microinjected with 50 μM *YAP* siRNA (treatment), water (sham control), or were left uninjected (control). MII oocytes in each group were then parthenogenetically activated and cultured to the blastocyst stage. At the 2-cell, 4-cell, and 8-cell stage, a subset of embryos from each group were isolated and subjected to qPCR to examine the relative expression of *YAP* mRNA. *YAP* siRNA injection significantly reduced the levels of *YAP* mRNA at the 2-cell, 4-cell, and 8-cell stages compared to the control groups (*p* < 0.05; Figure 2A). No differences in expression were observed between the sham injected and uninjected control groups.

Next, immunofluorescence confocal microscopy and western blotting analysis were used to determine the relative amount of YAP protein in embryos at the 2-cell, 4-cell, and morula stage. As shown in Figure 2B, the fluorescence signal of YAP protein in embryos injected with *YAP* siRNA was largely reduced at all three stages of development compared to the control groups. Consistent with these results, western blot analysis revealed that *YAP* siRNA significantly reduced the amount of YAP protein (*p* < 0.05; Figure 2C,D). Altogether, these results demonstrate that *YAP* siRNA can efficiently knockdown maternal *YAP* mRNA and protein in porcine early embryos.

### 3.3. YAP Knockdown Impedes Blastocyst Development and Perturbs Normal Lineage Allocation

We next sought to determine the biological role of maternal YAP in porcine preimplantation embryo development. To address this, *YAP* siRNA injected embryos and control embryos were cultured to the blastocyst stage and developmental rates were recorded. In a pilot experiment, we compared the developmental efficiency of embryos injected with either scrambled siRNA versus water. The results indicated that microinjection of scrambled siRNA or sham water did not affect cleavage or blastocyst rates compared to an uninjected control group (Supplementary Figure S2A,B). Thus, sham water injected embryos were used as negative controls in subsequent experiments.

**Figure 3:**
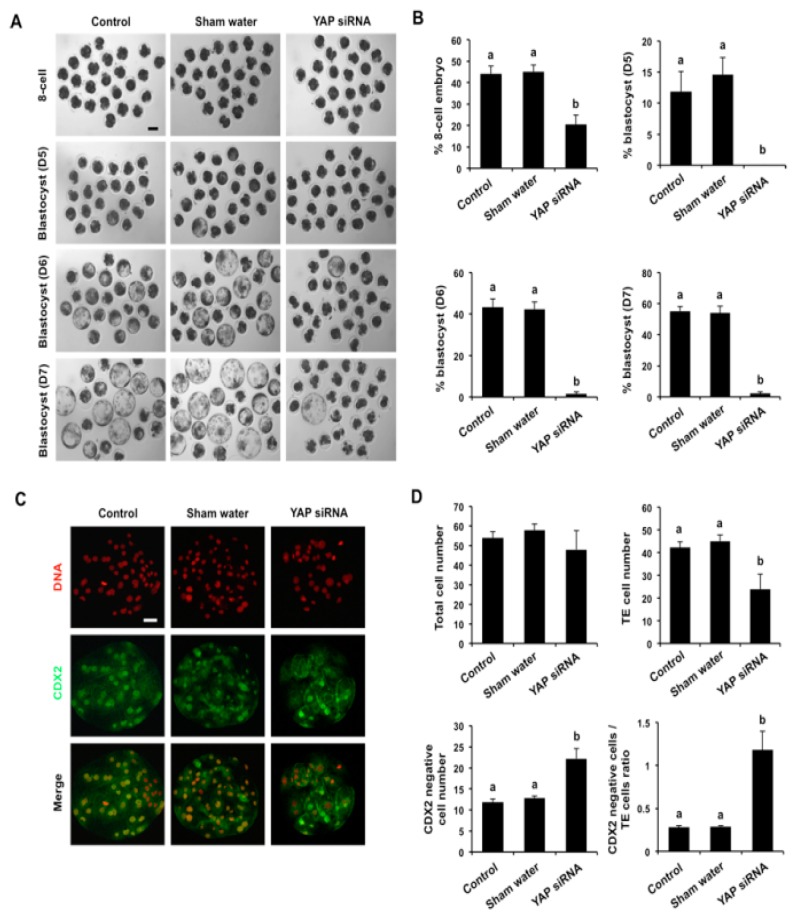
Effect of *YAP* knockdown on the developmental efficiency of porcine embryos. (**A**) Representative images of embryos at different stages. MII oocytes were microinjected with *YAP* siRNA. Uninjected oocytes or sham injected (water) served as two control groups. MII oocytes from each group were then parthenogenetically activated and cultured to the blastocyst stage. Scale bar: 100 µm. (**B**) Developmental rates of porcine preimplantation embryos. The rates of 8-cell embryos and blastocysts at day 5, 6, and 7 were recorded and statistically analyzed in each group. Data are expressed as mean ± S.E.M and different letters on the bars indicate significant differences (*p* < 0.05). (**C**) Immunofluorescence staining of blastocysts in each group using a CDX2 antibody. Blastocysts were stained to indicate CDX2 (green) and DNA (red). Representative images obtained using confocal microscopy are shown. The experiment was independently repeated three times with at least 10 blastocysts per group. The bottom panel in each group shows merged images between CDX2 and DNA. Scale bar: 50 µm. (**D**) Lineage allocation analysis of *YAP* knockdown and control blastocysts. Total cell numbers, TE cells, CDX2 negative cells, and the ratio of CDX2 negative cells to TE cells were separately recorded and subjected to statistical analysis. TE: trophectoderm. Data are represented as mean ± S.E.M and different letters on the bars indicate significant differences (*p* < 0.05).

In the next set of experiments, the developmental rates of *YAP* siRNA injected embryos were compared to sham injected and uninjected embryos. *YAP* knockdown had no effect on development to the 2-cell and 4-cell stage (Supplementary Figure S3A,B), however there was a significant reduction in embryos that developed to the 8-cell and blastocyst stages (Day 5–7) compared to controls (*p* < 0.05; Figure 3A,B and Supplementary Figure S3C). Importantly, a small percentage of *YAP* knockdown embryos developed to the blastocyst stage (Figure 3A). This allowed us to examine lineage allocation and further determine whether the quality of *YAP* knockdown blastocysts was impaired. To accomplish this, blastocysts from the treatment and control groups were stained with a CDX2 antibody to determine the TE cell number (Figure 3C). The number of CDX2 negative cells was indirectly determined by subtracting the TE number from the total cell number. The results showed that total cell number did not change between *YAP* knockdown and the control groups (Figure 3D). However, *YAP* knockdown resulted in a significant reduction in TE cell number and an increase in the CDX2 negative cell number (*p* < 0.05; Figure 3D). In addition, the ratio of CDX2 negative cells to TE cells in *YAP* knockdown blastocysts significantly increased compared to the control groups (*p* < 0.05; Figure 3D). To rule out potential interference by a copy of the paternal *YAP* gene, IVF embryos were used to further confirm the above observed developmental phenotypes. We found that *YAP* knockdown in IVF embryos also severely blocked blastocyst formation (*p* < 0.05; Supplementary Figure S4A,B). Together, these data indicate that maternal YAP is essential for porcine blastocyst development and normal lineage allocation.

### 3.4. YAP-Inhibited Embryos Recapitulate the Phenotypes of YAP Knockdown Embryos

**Figure 4:**
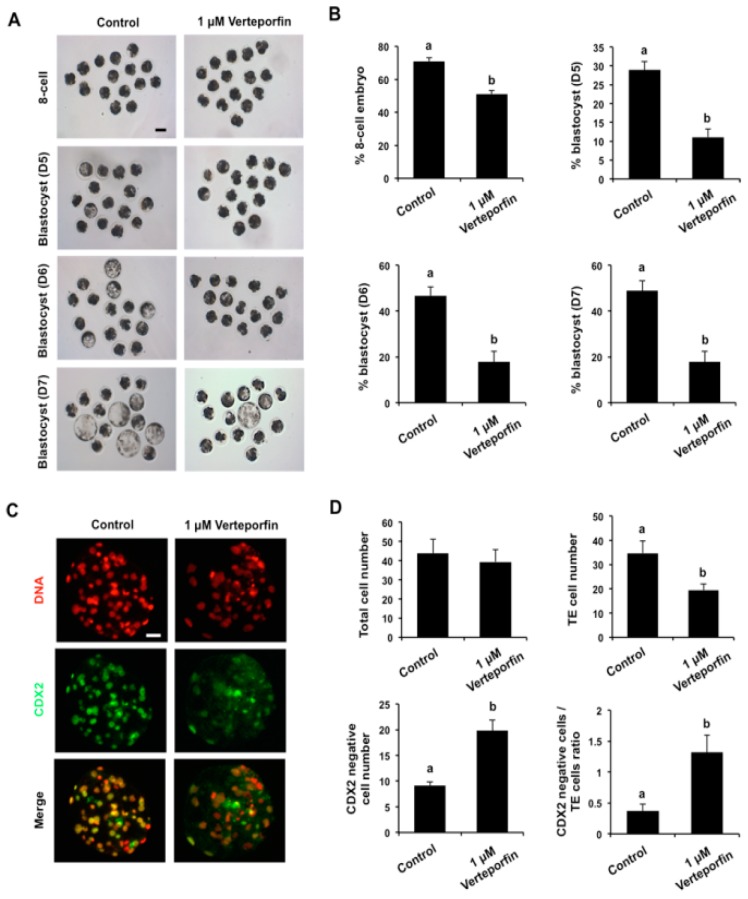
Effect of YAP inhibition on the developmental efficiency of porcine embryos. (**A**) Representative images of embryos at different stages from control and verteporfin treatment groups. One-cell embryos were cultured in vitro for 7 days in the presence of 1 μM verteporfin (YAP inhibitor) dissolved in DMSO. Embryos cultured in medium containing an equivalent amount of DMSO served as a control group. Scale bar: 100 µm. (**B**) The developmental rates of early embryos cultured with or without verteporfin. Developmental rates of 8-cell embryos and blastocysts on day 5, 6, and 7 were recorded in each group. Data are expressed as mean ± S.E.M and different letters denote significant differences (*p* < 0.05). (**C**) Representative fluorescence images of blastocysts stained with CDX2 antibody. Blastocysts were stained to indicate CDX2 (green) and DNA (red). The experiment was independently repeated three times with at least 10 blastocysts per group. The bottom panel in each group shows the merged images between CDX2 and DNA. Scale bar: 50 µm. (**D**) Lineage allocation analysis of YAP inhibited and control blastocysts. Total cell numbers, TE cells, CDX2 negative cells, and the ratio of CDX2 negative cells to TE cells were separately recorded and subjected to statistical analysis. TE: trophectoderm. Data are shown as mean ± S.E.M and different letters denote significant differences (*p* < 0.05).

In other cellular contexts, YAP interacts with TEAD family proteins to regulate target gene expression [14]. Therefore, we tested whether YAP function in porcine preimplantation embryos depended on interactions with TEAD proteins. One-cell embryos were cultured for 7 days in the presence of 1 μM verteporfin, a well-documented disruptor of YAP and TEAD protein interactions. We found that, similar to *YAP* knockdown, pharmacological inhibition of YAP and TEAD interactions did not impair development from the 2-cell to 4-cell stage, but significantly reduced the developmental efficiency of embryos that reached the 8-cell and blastocyst stages (*p* < 0.05; Figure 4A,B). Additionally, we stained the small proportion of embryos that formed blastocysts with CDX2 antibody to examine lineage allocation (Figure 4C). The results implied that inhibition of YAP/TEAD interactions also resulted in a reduction in TE cell number and increases in CDX2 negative cell numbers (*p* < 0.05; Figure 4D). The ratio of CDX2 negative cells to TE cells in the YAP inhibited blastocysts was significantly higher compared to that in control blastocysts (*p* < 0.05; Figure 4D).

To further unveil the temporal requirement for maternal YAP during porcine early embryo development, embryos were exposed to 1 μM verteporfin during two periods of time during preimplantation development. Embryos were treated between the 1-cell to 8-cell stage (day 0–3) and between the 8-cell to the blastocyst stage (day 3–7). As shown in Supplementary Figure S5A,B, YAP inhibition during day 0–3 or day 3–7 caused a similar reduction in blastocyst development compared to those induced by *YAP* knockdown, implying that YAP function is required for both early and later stages of preimplantation embryo development. Collectively, these data demonstrate that in pig embryos, YAP functions via TEAD interactions and is essential for normal blastocyst development and lineage allocation.

### 3.5. Maternal YAP Regulates the Expression of Genes Important for Lineage Commitment, TJ Assembly, and Fluid Accumulation

**Figure 5:**
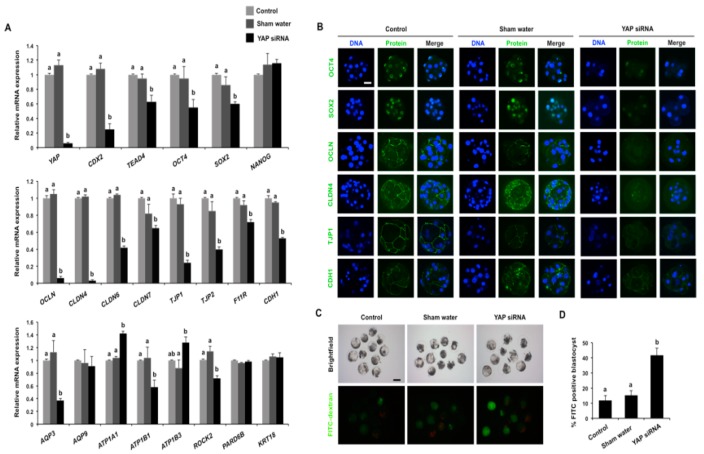
*YAP* knockdown perturbs the expression of genes required for lineage commitment, TJ assembly, and fluid accumulation. (**A**) Expression of putative YAP target genes in control and *YAP* knockdown morula. Relative expression of YAP target genes was determined by qPCR. Data were normalized against an endogenous reference gene (*EF1α1*) and the data from the control were set to 1. Data are shown as mean ± S.E.M and different letters denote significant differences (*p* < 0.05). (**B**) Expression and localization of YAP target gene proteins in control and *YAP* knockdown morula. Target proteins and DNA are represented as green and red, respectively. Representative images obtained using confocal microscopy are shown. The experiment was independently repeated three times with at least 15 morula per group. Scale bar: 100 µm. (**C**) Representative brightfield and fluorescence images of FITC-dextran treated blastocysts from the control and *YAP* knockdown groups. Blastocysts in each group were incubated in the medium containing 1 mg/mL 40 kDa FITC-dextran for 30 min and then the blastocysts were visualized under an inverted fluorescence microscope. Scale bar: 100 µm. (**D**) Analysis of paracellular permeability in trophectoderm by FITC-dextran uptake assay. The number of FITC positive blastocysts in each group was statistically analyzed. Data are shown as mean ± S.E.M and different letters denote significant differences (*p* < 0.05).

In mice, loss of YAP function results in epithelialization defects during the morula to blastocyst transition [18]. Because YAP knockdown and pharmacological inhibition in porcine embryos blocks blastocyst formation, we hypothesized that maternal YAP might be a critical transcriptional co-regulator of genes associated with blastocyst development. To test this, we performed qPCR in *YAP* knockdown and control embryos to determine the expression levels of 21 genes associated with blastocyst development (Figure 5A). We first examined the expression of lineage commitment genes, such as *CDX2*, *TEAD4*, *OCT4*, *SOX2*, and *NANOG*. These genes are important for blastocyst development in mice [20], pigs [21], and/or goats [22]. The expression levels of *CDX2*, *TEAD4*, *OCT4*, and *SOX2* were significantly reduced in *YAP* knockdown morula (*p* < 0.05; Figure 5A, upper panel), whereas the expression of *NANOG* mRNA was not affected in morula (Figure 5A, upper panel). Evaluation of these same five genes at the 8-cell stage revealed that their expression was not different between *YAP* knockdown and control embryos (Supplementary Figure S6), indicating that YAP regulates these lineage commitment genes after the 8-cell stage during the morula to blastocyst transition.

To further uncover the molecular mechanisms underlying the developmental phenotypes of *YAP* knockdown embryos, we examined the expression of genes required for TJ assembly and fluid accumulation, which are critical for both paracellular sealing of the TE epithelium and formation of blastocoel cavity [11]. We observed a significant reduction in the expression levels of *OCLN*, *CLDN4*, *CLDN6*, *CLDN7*, *TJP1*, *TJP2*, *F11R*, and *CDH1* in *YAP* knockdown embryos (*p* < 0.05; Figure 5A, middle panel). In addition, we examined the expression levels of *AQP3*, *APQ9, ATP1B1, ATP1A1,* and *ATP1B3;* these genes are important for the accumulation of fluid within the blastocoel cavity by establishment of a trans-TE ionic gradient [10,11]. We found that the levels of *AQP3* and *ATP1B1* mRNA significantly decreased, whereas the expression levels of *ATP1A1* and *ATP1B3* increased in *YAP* knockdown embryos (*p* < 0.05; Figure 5A, bottom panel). *YAP* knockdown did not affect the expression of *APQ9* mRNA. In addition, we examined the expression of genes related to cell polarity and cytoskeleton that are involved in the regulation of blastocyst development in mice [12,23]. The results revealed that the expression levels of *ROCK2* were significantly reduced (*p* < 0.05), whereas *PARD6B* and *KRT18* expression was not significantly affected in *YAP* knockdown embryos (Figure 5A, bottom panel).

Based on the qPCR data above, we examined the expression and localization of the corresponding proteins for a subset of genes that were downregulated in *YAP* knockdown embryos. We only selected commercially available antibodies which work well in porcine embryos. These included OCT4, SOX2, OCLN, CLDN4, TJP1, and CDH1. Consistent with the qPCR data, the abundance of the corresponding proteins was also dramatically reduced in *YAP* knockdown embryos compared to controls. The lineage commitment proteins OCT4 and SOX2 were widely reduced in YAP knockdown morulae (Figure 5B). Likewise, the apical and basolateral localized proteins such as OCLN, CLDN4, TJP1, and CDH1 were severely diminished and not visible in *YAP* knockdown embryos compared to controls (Figure 5B).

TJ complexes mediate paracellular sealing between apical and basolateral domains of the TE epithelium to facilitate blastocoel cavity formation [10]. Given that *YAP* knockdown led to downregulation of key TJ proteins, we hypothesized that *YAP* knockdown impaired the integrity of these complexes and disrupted paracellular sealing. To directly test this, we examined the permeability of TJ complexes in porcine blastocysts by the FITC-dextran (40 kDa) exclusion test. The results revealed that the fluorescence intensity of *YAP* knockdown blastocysts and the percentage of FITC-positive blastocysts in the *YAP* knockdown group were significantly higher than that in the control groups (Figure 5C,D), suggesting that the barrier function of the TE epithelium was impaired in *YAP* knockdown embryos. Altogether, these results demonstrate that maternal YAP is required for the correct expression of genes that are essential for the establishment of a functional TE epithelium.

### 3.6. YAP^+^ Blastomeres Complement YAP Knockdown Blastomeres to Sustain Blastocyst Development

**Figure 6:**
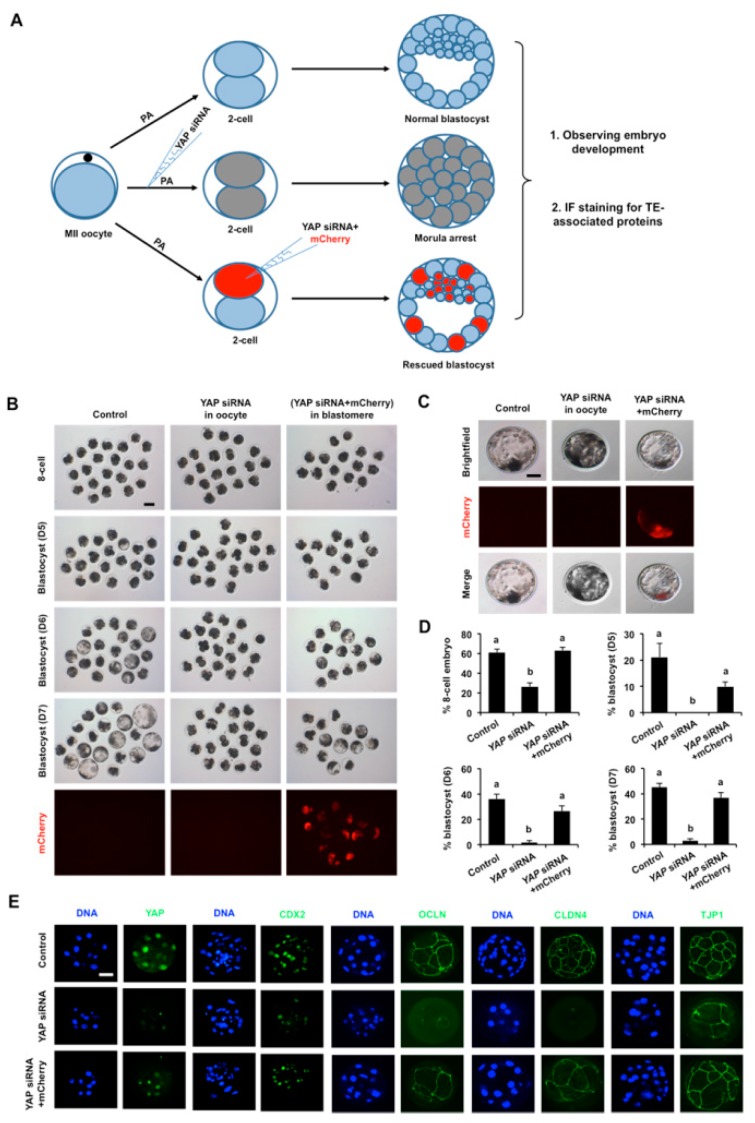
*YAP^+^* blastomeres complement *YAP* deleted blastomeres to sustain blastocyst development. (**A**) Experimental design describing *YAP* knockdown rescue experiments in embryos. PA: parthenogenetic activation, TE: trophectoderm. (**B**) Representative images of embryos at different stages from control, *YAP* knockdown in oocytes and *YAP* knockdown in single blastomere of 2-cell embryos. MII oocytes were microinjected with *YAP* siRNA. Single blastomere of a 2-cell embryo was co-microinjected with both *YAP* siRNA and mCherry mRNA. Uninjected MII oocytes served as a control. Embryos in each group were cultured until the blastocyst stage. The blastocysts were then visualized under an inverted fluorescence microscope. (**C**). Enlarged images of single blastocysts from each group is shown. Scale bar: 50 µm. (**D**) The developmental rates of early embryos. Proportion of embryos that developed to the 8-cell stage and blastocysts on day 5, 6, and 7 were recorded. Data are expressed as mean ± S.E.M and different letters denote significant differences (*p* < 0.05). (**E**) Expression and localization of both YAP and its target proteins in morula. Target proteins were evaluated using specific antibodies (green) and DNA was visualized using propidium iodide (red). Representative images obtained using confocal microscopy are shown. The experiment was independently repeated three times with at least 15 morula and blastocysts per group. Scale bar: 50 µm.

To further examine the role of YAP in the TE, a series of single cell *YAP* knockdown experiments were performed in 2-cell embryos. We hypothesized that the cellular progeny of the uninjected YAP^+^ blastomere would complement the *YAP* knockdown blastomeres to restore features of the TE, which in turn, would sustain blastocyst development. As shown in Figure 6A, single blastomeres in 2-cell embryos were co-injected with *YAP* siRNA and histone mCherry mRNA (co-injection group) and embryos were cultured until the blastocyst stage. In one set of controls, YAP *siRNA* was injected into MII oocytes, then the oocytes were activated and cultured to the blastocyst stage. In a second set of controls, uninjected 2-cell embryos were cultured to the blastocyst stage. As expected, the developmental rates of MII oocytes injected with *YAP* siRNA were significantly reduced and a large proportion of embryos arrested at the 8-cell and morula stages (Figure 6B,D). In contrast, 2-cell embryos co-injected with *YAP* siRNA and histone mCherry mRNA in a single blastomere developed to the blastocyst stage at a rate that was similar to uninjected 2-cell embryos (Figure 6B,D), suggesting that the cellular descendants of the YAP^+^ uninjected blastomere rescued blastocyst formation. Closer examination of the YAP^−^/mCherry^+^ blastomeres in blastocysts stained with a CDX2 antibody revealed a random distribution of the YAP^−^/mCherry^+^ blastomeres in CDX2 positive and CDX2 negative cells (Figure 6C, Supplementary Figure S7). This suggests that *YAP* knockdown did not alter the developmental fate of blastomeres in blastocysts.

Next, we tested whether key characteristics of the TE epithelium were restored in 2-cell embryos co-injected with *YAP* siRNA and histone mCherry mRNA. Morula and/or blastocysts from each group were subjected to immunofluorescence confocal microscopy using antibodies for YAP, CDX2, OCLN, CLDN4, and TJP1 (Figure 6E). We observed that the expression and localization of these proteins were largely restored in both the nucleus and membrane of these blastocysts, indicating that a functional TE epithelium was successfully established. Altogether, these data indicate that in 2-cell embryos injected with *YAP* siRNA, the progeny of the uninjected YAP^+^ blastomere are sufficient to rescue blastocyst formation.

## 4. Discussion

**Figure 7:**
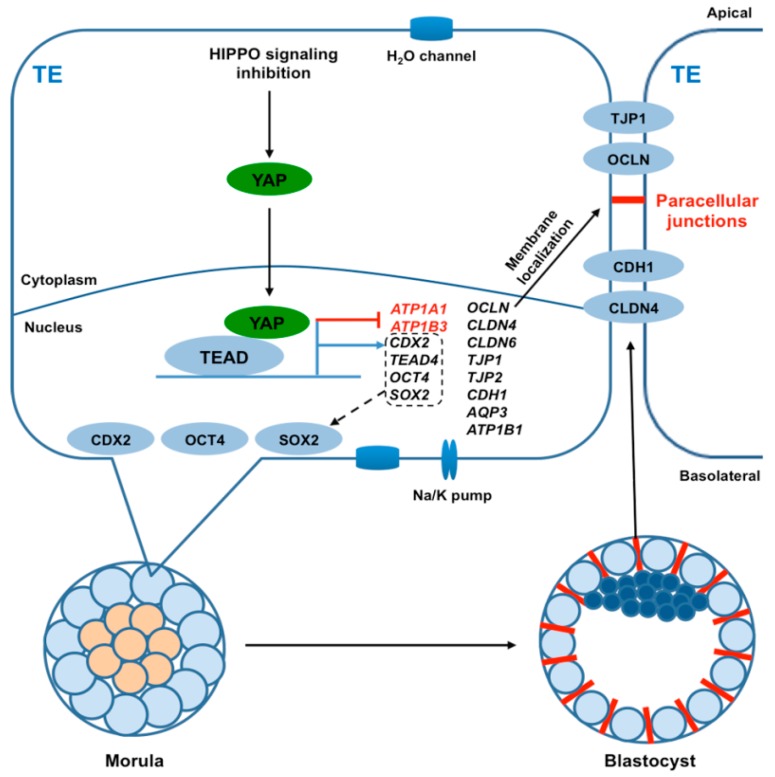
Working model illustrating how maternal YAP regulates trophectoderm integrity to facilitate porcine blastocyst development. In the TE epithelium, inactivation of hippo signaling induces the translocation of cytoplasmic YAP into the nucleus, which in turn binds to TEAD family proteins to form a transcriptional complex. The YAP-containing complex positively regulates the expression of genes (black) that are important for lineage commitment (*CDX2*, *TEAD4*, *OCT4*, and *SOX2*), TJ assembly (*OCLN*, *CLDN4*, *CLDN6*, *CDH1*, *TJP1*, and *TJP2*), and fluid accumulation (*ATP1B1* encoding Na/K-ATPase, *AQP3* encoding H_2_O transporter). The complex also negatively regulates the expression of two genes (red) encoding Na/K-ATPase (*ATP1A1*, *ATP1B3*). Collectively, YAP is necessary for the establishment of TJ junction complexes between TE cells and Na/K pumps and H_2_O pumps between the apical domain and basolateral domains to promote paracellular sealing and blastocoel formation.

In the present study, we demonstrated that *YAP* mRNA is a maternally derived transcript in pig embryos and is required for normal blastocyst development. In contrast, in mice YAP is both maternally and zygotically expressed and is required for both genome activation and blastocyst development [19]. Our data in pig oocytes and embryos indicates that maternal YAP promotes blastocyst formation through co-regulation of key genes that are important for lineage commitment, TJ assembly, and fluid accumulation. The correct expression of these genes is important for proper lineage allocation and paracellular sealing. Therefore, we propose a working model in which maternally derived YAP cooperates with TEAD family proteins to promote porcine blastocyst development through transcriptional co-regulation of key genes that are essential for lineage commitment, tight junction assembly, and fluid accumulation (Figure 7).

Lineage allocation in mammalian blastocysts is mediated by key lineage commitment TFs such as CDX2, TEAD4, OCT4, and SOX2 [24,25,26,27]. In this study, we found that RNAi mediated the knockdown of maternal YAP severely perturbed lineage allocation in pig blastocysts. The ratio of the CDX2 negative cell number to TE cell number was significantly increased. Moreover, during the morula to blastocyst transition, the expression of CDX2, TEAD4, OCT4, and SOX2 were downregulated in *YAP* knockdown embryos, indicating that maternal YAP is necessary for their proper expression. Consistent with our findings, studies in mice showed that YAP and TEAD4-mediated co-regulation of CDX2 and SOX2 is implicated in the specification of TE and epiblast lineages [16,28]. Importantly, in our study treatment of pig embryos with verteporfin, a specific inhibitor of YAP and TEAD4 interactions also disrupted lineage allocation and phenocopied YAP knockdown embryos, indicating that YAP-TEAD4 interactions are critical for lineage allocation in both pigs and mice.

A recent study in mice revealed that preimplantation embryos lacking maternal and zygotic YAP exhibited serious epithelialization defects [18], implying that YAP may also regulate the expression of TE genes that are important for TJ assembly and blastocoel formation. Accordingly, we identified a number of genes important in TJ assembly (*OCLN*, *CLDN4*, *CLDN6*, *CLDN7*, *TJP1*, *TJP2*, and *F11R*), adherens junction formation (*CDH1*), and fluid accumulation (*AQP3* and *ATP1B1)*. A similar set of genes was identified in transcription factor AP-2 gamma (*TFAP2C)* knockdown mouse embryos [29], indicating that in mice and pigs, TFAP2C and YAP may regulate a similar group of genes. Previous studies demonstrated that claudin family proteins regulate TJ assembly and paracellular sealing in epithelial cells [30]. The inhibition of OCLN, CLDN4, and CLDN6 protein by neutralizing antibody or an inhibitory peptide blocked blastocyst formation in mice [31,32]. Furthermore, *CLDN7* knockdown impairs blastocyst development in pigs (unpublished data). TJ transmembrane proteins, such as TJP1, TJP2, and F11R, and adherens junction proteins, such as CDH1, are also essential for blastocyst development in mice [33,34,35,36], indicating that these proteins play a conserved role in mice and pigs. Lastly, *AQP3* or *ATP1B1* play an important role in blastocoel formation by regulating the accumulation of fluid. In mouse embryos, RNAi mediated knockdown of *Aqp3* and *Atp1b1* blocks blastocyst formation [37,38]. Altogether, these results demonstrate that maternal YAP in pig embryos promotes TE development via the regulation of key genes involved in TJ assembly and blastocoel formation. Future chromatin immunoprecipitation (ChIP) studies in pig embryos will be necessary in order to demonstrate that YAP is a direct transcriptional regulator of these gene families.

Consistent with the altered expression of genes involved in TJ assembly and blastocoel formation, we found that the permeability of TE epithelium was impaired in *YAP* knockdown embryos. Functional inhibition of claudin proteins such as OCLN, CLDN4, and CLDN6 [32] in mouse embryos and knockdown of *CLDN7* in pig embryos (unpublished data) disrupts paracellular sealing. This suggests that maternal YAP regulates key TJ genes required for paracellular sealing. Importantly, RNAi experiments in 2-cell embryos revealed that uninjected blastomeres (YAP+) could complement *YAP* knockdown blastomeres and promote blastocyst development via TJ gene expression. Indeed, the expression and localization of TE related proteins were restored at the TE. Altogether, our results demonstrate that in pig embryos, maternal YAP is a key factor required for TJ assembly and paracellular sealing.

In conclusion, our findings demonstrate that maternal YAP facilitates porcine blastocyst development through transcriptional regulation of key genes that are essential for lineage commitment, tight junction assembly, and fluid accumulation. In pig preimplantation embryos, YAP appears to play a much larger role in TJ assembly, paracellular sealing, and fluid accumulation, which are key events that are important for embryo attachment, placentation, and development to term. Our results may provide new insights into why IVP pig embryos exhibit low developmental competence. Future research studies will focus on the development of novel strategies for improving the developmental competence of IVP embryos.

## Supplementary Materials

The following are available online at https://www.mdpi.com/2073-4409/8/12/1606/s1. Figure S1: Validation of the specificity of a subset of primary antibodies used in this study; Figure S2: Effect of scrambled siRNA on early cleavage development of porcine parthenogenetically activated embryos; Figure S3: Effect of *YAP* knockdown on early cleavage development of porcine parthenogenetically activated embryos; Figure S4: Effect of *YAP* knockdown on early development of embryos produced by in vitro fertilization; Figure S5: YAP activity is temporally required for embryonic development before and after the 8-cell stage; Figure S6: *YAP* knockdown does not alter the expression of genes essential for commitment of the first two lineages at the 8-cell stage; Figure S7: Effect of *YAP* knockdown on the localization of blastomeres in blastocysts; Table S1: Information on YAP siRNA sequences; Table S2: Porcine-specific primer sequences used in this study; Table S3: Information on primary and secondary antibodies used in this study.
