# Supplementary material for: Maternal Yes-Associated Protein Participates in Porcine Blastocyst Development via Modulation of Trophectoderm Epithelium Barrier Function
Source: Cells. 2019 Dec 11;8(12):1606. doi: 10.3390/cells8121606 (PMC6952962; doi:10.3390/cells8121606)
Supplement: Supplementary file 1 [file cells-08-01606-s001.pdf]

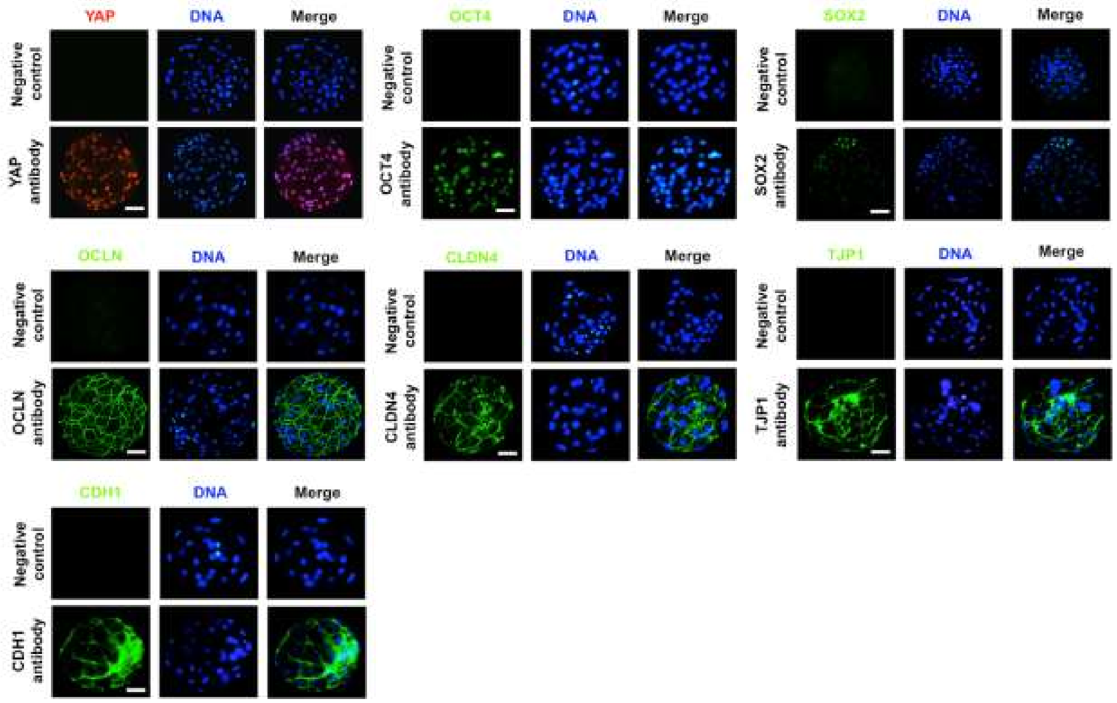

Figure S1. Validation of the specificity of partial primary antibodies used in this study. Commercially available primary antibodies against YAP, OCT4, SOX2, OCLN, CLDN4, TJP1, and CDH1 were tested on porcine blastocysts. In a subset of embryos, the primary antibody was omitted and the embryos were treated with serum alone. These embryos served as a negative control. Shown are representative images obtained using confocal microscopy. Scale bar: 50 μm.

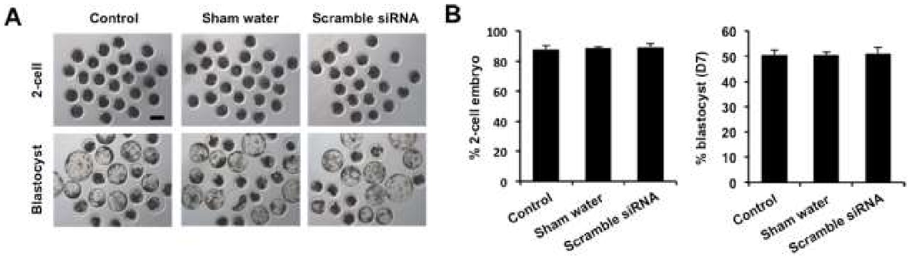

Figure S2. Effect of scrambled siRNA on early cleavage development of porcine parthenogenetically activated embryos. (**A**) Representative images of embryos from control, sham injected (water) and scrambled siRNA injected group. Scale bar: 100 μm. (**B**) The rates of 2-cell and blastocyst development in each group were recorded and statistically analyzed. Data are expressed as mean ± S.E.M.

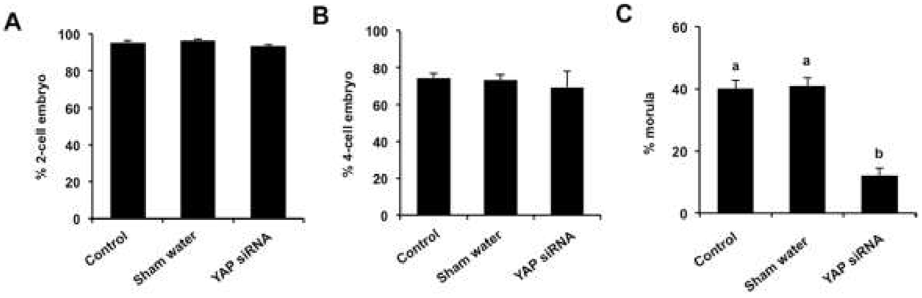

Figure S3. Effect of *YAP* knockdown on early cleavage development of porcine parthenogenetically activated embryos. The developmental rates of 2-cell, 4-cell, and morula stage embryos in each group were recorded and statistically analyzed. Data are expressed as mean ± S.E.M and different letters denote significant differences ($P < 0.05$).

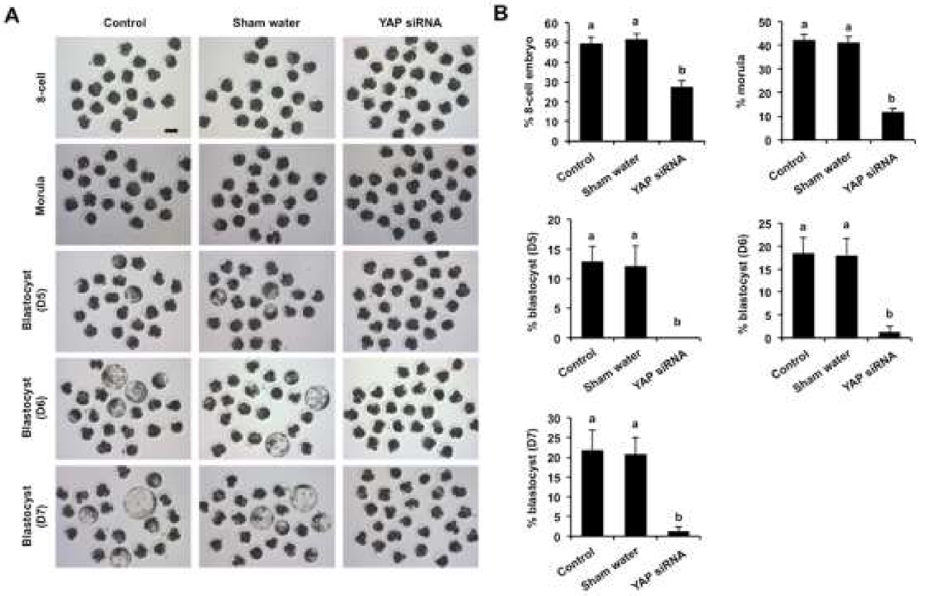

Figure S4. Effect of *YAP* knockdown on early development of embryos produced by *in vitro* fertilization. (**A**) Representative images of embryos derived from *in vitro* fertilization (IVF) at different stages. MII oocytes were co-incubated with sperm to produce zygotes. Putative zygotes were microinjected with *YAP* siRNA. Uninjected zygotes or sham injected (water) served as two control groups. Zygotes in each group were cultured up to the blastocyst stage. Scale bar: 100 μm. (**B**) The developmental rate of early IVF embryos. The rates of 8-cell, morula, and blastocysts at day 5, 6, 7 were recorded and statistically analyzed in each group. Data are expressed as mean ± S.E.M and different letters denote significant differences ($P < 0.05$).

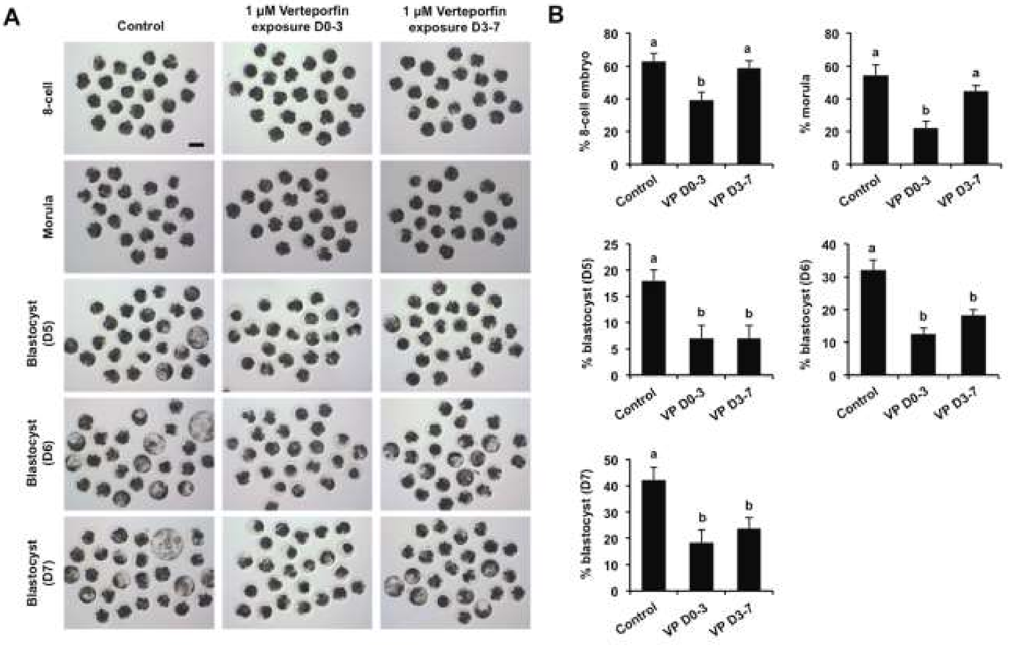

Figure S5. YAP activity is temporally required for embryonic development before and after 8-cell stage. (**A**) Representative images of embryos derived from parthenogenetic activation at different stages. MII oocytes were parthenogenetically activated and cultured up to the blastocyst stage. Embryos were exposed to the indicated concentration of YAP inhibitor (verteporfin) from the 1-cell to the 8-cell stage (day 0-3) or the 8-cell to the blastocyst stage (day 3-7). Embryos without any treatment serve as a control group. VP: verteporfin. Scale bar: 100 μm. (**B**) The developmental rate of early embryos. The rates of 8-cell, morula and blastocysts at day 5, 6, 7 were recorded and statistically analyzed in each group. Data are expressed as mean ± S.E.M and different letters on the bars indicate significant differences ($P < 0.05$).

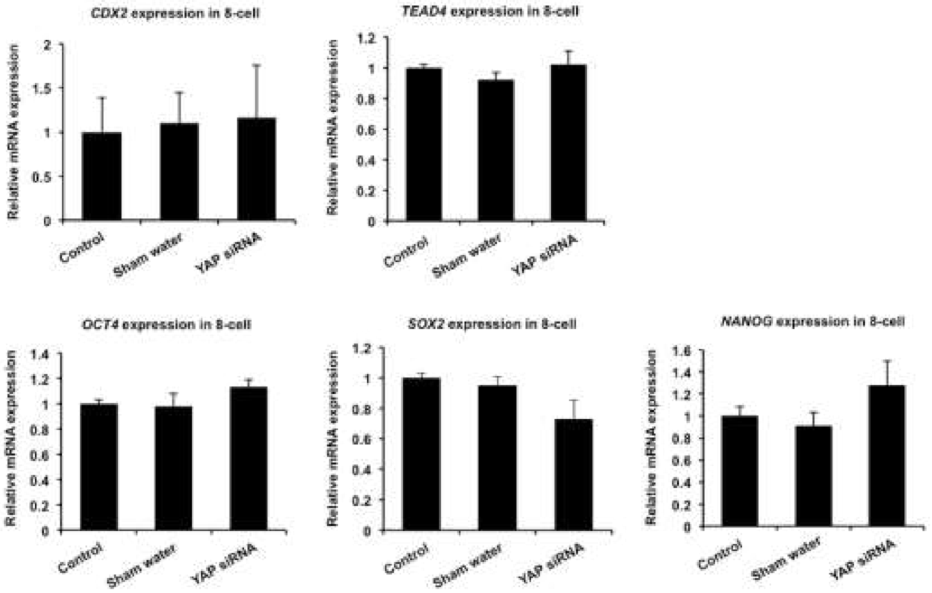

Figure S6. *YAP* knockdown does not alter expression of genes essential for commitment of the first two lineages at 8-cell stage. Relative expression of *CDX2, TEAD4, OCT4, SOX2, and NANOG* in 8-cell embryos from control, sham injected (water), and siRNA injection was analyzed by qPCR. Data were normalized against endogenous reference gene *EF1α1* and the data from each stage were relative to control group. Data are shown as mean ± S.E.M and different letters on the bars indicate significant differences ($P < 0.05$).

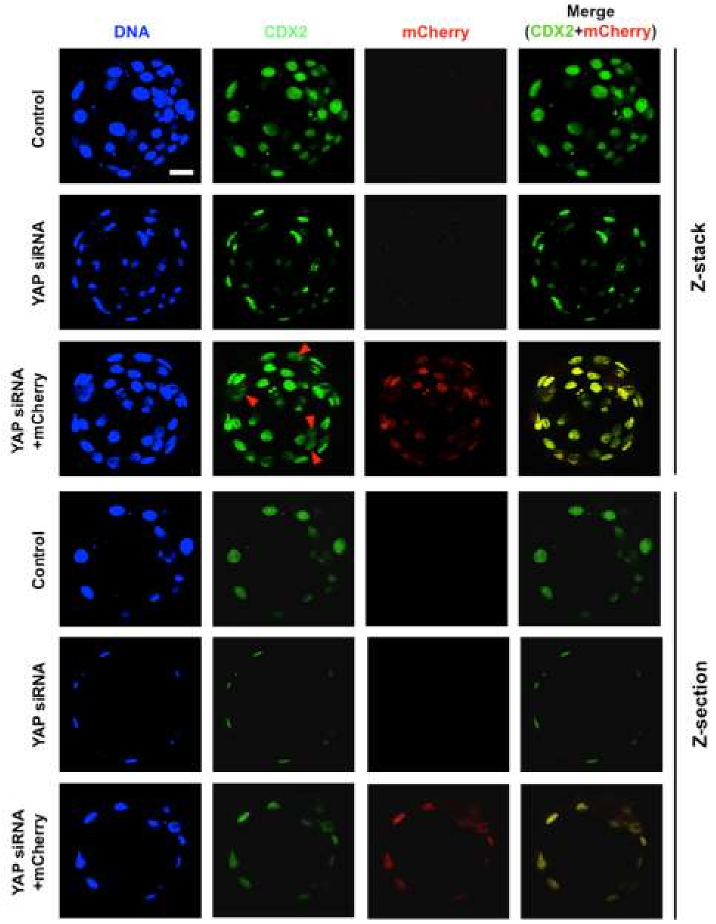

Figure S7. Effect of *YAP* knockdown on the localization of blastomeres in blastocysts. Representative Z-stack and Z-section images of blastocysts from control, *YAP* knockdown in oocytes, and *YAP* knockdown in single blastomeres in 2-cell embryos. Blastocysts were stained for CDX2 (green) and DNA (blue). mCherry positive blastomeres were labeled with red. The experiment was independently repeated three times with at least 10 blastocysts per group. Arrowheads indicate CDX2 positive and mCherry negative blastomeres. Scale bar: 50 μm.
